# Food Addiction and Eating Addiction: Scientific Advances and Their Clinical, Social and Policy Implications

**DOI:** 10.3390/nu12051485

**Published:** 2020-05-20

**Authors:** Adrian Carter, Charlotte A. Hardman, Tracy Burrows

**Affiliations:** 1 School of Psychological Sciences and the Turner Institute for Brain and Mental Health, Monash University, Clayton, VIC 3800, Australia; 2 Department of Psychology, University of Liverpool, Liverpool L69 7ZA, UK; charlotte.hardman@liverpool.ac.uk; 3 School of Health Sciences, Faculty of Health, University of Newcastle, Newcastle, NSW 2308, Australia; tracy.burrows@newcastle.edu.au

There is a growing understanding within the literature that certain foods, particularly those high in refined sugars and fats, may have addictive potential for some individuals. Moreover, individuals who are overweight and have obesity display dietary intake patterns that resemble the ways in which individuals with substance use disorders consume addictive drugs. While food addiction is not yet recognized in the Diagnostic and Statistical Manual of Mental Disorders, Fifth Edition (DSM-5), there are many similarities with substance use disorders, and a growing acceptance that some forms of obesity should be treated as a food addiction. Despite growing research in this area, there remain many unresolved questions about the science of food addiction and its potential impact upon: how we treat overweight and overeating; stigmatization and discrimination of people who are overweight; internalized weight bias and treatment seeking; as well as policies to reduce excess weight and overeating.

This interdisciplinary special issue collects 10 articles, including reviews and original research, that further our understanding and application of the science of addictive eating. These papers span a broad range of areas, including basic science, clinical assessment tools, neural responses to addictive foods, as well as insights into future treatments and public health policies, and the possible stigma associated with food addiction.

## 1. Validation of Food Addiction Scales

### Validation of the Japanese Version of the Yale Food Addiction Scale 2.0 (J-YFAS 2.0)

The Yale Food Addiction Scale (YFAS) is the most widely used diagnostic tool for food addiction, and has been translated into numerous languages, including Italian, French, German, Spanish, Arabic Chinese and Turkish. In this special issue, Khine and colleagues [1] describe the translation and validation of the Japanese version of the Yale Food Addiction Scale 2.0 (J-YFAS 2.0), carried out in 731 undergraduate students. The J-YFAS 2.0 has a one-factor structure and adequate convergent validity and reliability, similar to the YFAS 2.0 in other languages. Prevalence of J-YFAS 2.0-diagnosed mild, moderate, and severe food addiction was 1.1%, 1.2%, and 1.0% respectively.

## 2. Neural Responses Underlying Food Addiction

### 2.1. Food Addiction Symptoms and Amygdala Response in Fasted and Fed States

Pursey et al. [2] conducted a small pilot study to explore the association between food addiction symptoms and activation in the basolateral amygdala and central amygdala. 12 females, aged 24.1 ± 2.6 years, completed two functional magnetic resonance imaging (fMRI) scans (fasted and fed) while viewing high-calorie food images and low-calorie food images. Food addiction symptoms were assessed using the Yale Food Addiction Scale. Participants had a mean BMI of 27.4 ± 5.0 kg/m^2^, and food addiction symptom score of 4.1 ± 2.2. The results found a significant positive association, between food addiction symptoms, and higher activation of the left basolateral amygdala to high-calorie versus low-calorie foods in the fasted session, but not the fed session. There were no significant associations with the central amygdala in either session. 

### 2.2. Increasing Chocolate’s Sugar Content Enhances Its Psychoactive Effects and Intake

This study by Caperson and colleagues explored the potential psychoactive effect of chocolate [3]. Participants consumed 5 g of a commercially available chocolate with increasing amounts of sugar (90% cocoa, 85% cocoa, 70% cocoa, and milk chocolate). After each chocolate sample, participants completed the Psychoactive Effects Questionnaire (PEQ) and the Binge Eating Scale (BES). Participants were also allowed to eat as much as they wanted of each of the different chocolates. Casperson et al. [3] found that the excitement subscale of the PEQ increased (relative to baseline) after the 90% cocoa. The Morphine–Benzedrine Group subscale (containing questions about wellbeing and euphoria) and the Morphine subscale (focusing on attitudes and physical sensations) increased after the 85th cocoa sample. This suggests incremental increases in the sugar content of chocolate has a psychoactive effect which enhances the addictive-like eating response. 

## 3. Implications for Treatment

### 3.1. Food Addiction: Implications for the Diagnosis and Treatment of Overeating

The validity of a food addiction diagnosis remains controversial, despite a growing body of preclinical, neurobiological and clinical evidence supporting it. This literature review discusses the DSM-5 diagnostic criteria for substance use disorders, to summarize evidence for food addiction. Adams and colleagues [4] concluded that there is evidence to suggest that, for some individuals, food can induce addictive-type behaviors similar to those seen with other addictive substances. However, as several DSM-5 criteria have limited application to overeating, they argue that the term ‘food addiction’ is likely to apply only in a minority of cases. Research investigating the underlying psychological causes of overeating within the context of food addiction has also led to some novel treatment approaches, such as cognitive training tasks and neuro-modulation interventions.

### 3.2. Food Addiction in Eating Disorders and Obesity: Analysis of Clusters and Implications for Treatment

This study by Jimenez-Murcia et al. [5] identified three distinct clusters of food addiction in those with eating disorders and obesity. The study was conducted in 234 participants who scored positive on the Yale Food Addiction Scale 2.0. Cluster 1, classified as “dysfunctional”, was associated with the highest prevalence of other specified feeding or eating disorders and bulimia nervosa, as well as the highest eating disorder severity and psychopathology, and more dysfunctional personality traits. Cluster 2, classified as “moderate”, was associated with high prevalence of bulimia nervosa and binge eating disorders, and moderate levels of eating disorder psychopathology. Cluster 3, classified as “adaptive”, was characterized by high prevalence of obesity and binge eating disorders, low levels of eating disorder psychopathology and more functional personality traits. The authors suggest that the identification of types of food addiction traits may allow for more personalized treatment to improve outcomes. 

### 3.3. Food Addiction Is Associated with Irrational Beliefs via Trait Anxiety and Emotional Eating

Irrational beliefs are believed to be one of the prime causes of psychopathologies, including anxiety and depression. A study of 239 adults by Nolan and colleagues [6] investigated whether food addiction and emotional eating are associated with irrational beliefs. Questionnaires measuring food addiction, irrational beliefs, emotional eating, depression, trait anxiety, and anthropometry were assessed and reported. They found that irrational beliefs were significantly positively correlated with food addiction, emotional eating, depression and trait anxiety. Results also showed that irrational beliefs were associated with higher food addiction via higher trait anxiety and emotional eating acting in a serial pathway. As such, targeting irrational beliefs as a treatment in individuals who experience food addiction and emotional eating may be a reasonable approach for clinicians.

### 3.4. Fat Addiction: Psychological and Physiological Trajectory

A number of recent studies have attempted to parse out the psychological and physiological etiology of food addiction. This review article by Sarkar et al. [7] examines the specific role of dietary fats in compulsive overeating. They review preclinical, psychological and clinical evidence to argue for the addiction to fat rich diets as a prominent subset of food addiction. They then discuss the clinical implications of “fat addiction” for society. 

## 4. Associations between Food Addiction, Stigma and Public Policy

### 4.1. Ethical, Stigma, and Policy Implications of Food Addiction: A Scoping Review 

This scoping review by Cassin and colleagues [8] examines the potential ethical, stigma and health policy implications of food addiction described in the current literature. Their findings suggest that the literature on potential ethical implications was mostly focused on debates regarding individualized responsibility and sources for blame. Potential stigma focused on evidence of internalized and externalized stigma when food addiction is used as the explanation for obesity. The policy implications of food addiction largely drew on comparisons with the historic regulation of the tobacco industry to manage food addiction in policy, and the current challenges in classifying foods in terms of their addictive potential. 

### 4.2. Obesity Stigma: Is the ‘Food Addiction’ Label Feeding the Problem? 

There is significant debate around whether describing someone as addicted to food would increase or decrease weight-based stigma. Ruddock et al. [9] examined the effect of the food addiction label on stigmatizing attitudes towards an individual with obesity, and towards people with obesity more generally (i.e., general stigma). They presented the results of two online studies, where participants (n = 439, n = 523) read a short description about a woman described as ‘very overweight’. They found that a food addiction label may exacerbate stigmatizing attitudes towards an individual with obesity. However, the label appears to have no effect on general weight-based stigma. Stigmatizing attitudes towards people with obesity also appeared to be more pronounced in individuals with low levels of addiction-like eating behaviors, compared to high levels of addiction-like eating.

### 4.3. The Effect of a Food Addiction Explanation Model for Weight Control and Obesity on Weight Stigma

In the final paper of this special issue, O’Brien and colleagues [10] reported on two experimental studies examining the impact of a food addiction model of obesity and weight control on weight stigma. In both experiments, participants were randomized to receive one of two newspaper articles: one describing obesity as the result of a brain-based food addiction, and the other describing obesity as the result of diet and exercise. The food addiction explanation for weight control and obesity did not increase weight stigma, and resulted in lower stigma than the diet and exercise explanation, which attributes obesity to personal control. Their findings highlight the need for evidence-based health messaging about the causes of obesity, and the need for communications that do not exacerbate weight stigma.
